# Comparison of two postoperative complication grading systems following an elective orthopedic procedure in dogs

**DOI:** 10.3389/fvets.2026.1867094

**Published:** 2026-08-12

**Authors:** Savannah K. Crowdis, Faolain M. Barrett, Yu-An Guan, Justin J. Marrs, Derek B. Fox

**Affiliations:** Department of Veterinary Medicine and Surgery, College of Veterinary Medicine, University of Missouri, Columbia, MO, United States

**Keywords:** adapted Clavien-Dindo grading system, Cook grading system, dogs, postoperative complications, tibial plateau leveling osteotomy, veterinary orthopedics

## Abstract

**Introduction:**

A standardized system for grading postoperative complications in veterinary orthopedics was proposed in 2010 by Cook et al. categorizing complications as minor, major, or catastrophic. Despite broad acceptance, complication rates still vary widely between studies. An alternative to the Cook system has been proposed; the adapted Clavien-Dindo (aCD) system, which categorizes five complication grades and distinguishes them from two other outcomes: ‘sequelae’ and ‘failures-to-cure’. The objective of this study was to compare postoperative complication rates in dogs undergoing tibial plateau leveling osteotomies (TPLO) using both systems. We hypothesized that there would be no significant difference in complication rates between grading systems.

**Methods:**

Dogs undergoing TPLO between December 2023 and January 2025 were prospectively enrolled. Owner consent was obtained to review medical records and perform serial surveys for potential postoperative complications. A single investigator graded events with the Cook and aCD systems. Complication rates were compared with McNemar’s χ2 test (*α* < 0.05).

**Results:**

Eighty-five TPLO procedures in 79 dogs were evaluated. Using the aCD system, the complication rate was 28.2% while the Cook system reported 54.1%. McNemar’s χ2 test showed a significant difference (*p* < 0.001) between systems. Twenty-six percent of Cook-classified complications were considered non-complications by the aCD system.

**Discussion:**

We rejected our hypothesis that the Cook and aCD grading systems would yield comparable postoperative complication rates. The Cook system classified approximately twice the number of events as complications compared to the aCD system. A grading system with greater categorical resolution that distinguishes true complications from expected outcomes may provide a more clinically relevant assessment of postoperative risk.

## Introduction

Documenting postoperative complications is essential for determining the safety and efficacy of any surgical procedure. Although definitions vary slightly among grading systems, postoperative complications are generally considered deviations from the expected postoperative course that prompt clinical concern. Different grading systems of postoperative complications have been studied extensively in human-based medical research. A recent systematic review demonstrated that accurate, standardized, and reproducible grading systems are essential for tracking surgical outcomes (1). Such systems also facilitate comparisons across procedures and institutions and prioritize correlation between complication severity and clinical outcomes (1). Specifically, the Clavien-Dindo system has seen wide acceptance in human-based research with particular advantages in clinical settings (1, 2). The Clavien-Dindo system was developed to replace subjective descriptors such as ‘*minor*’ and ‘*major*’ with a reproducible, therapy-based severity scale, thereby enhancing comparability and consistency (3). To address the need for standardization in describing postoperative complications in veterinary medicine, Cook and colleagues developed a grading system that defined different levels of complications based on perceived clinical severity and impact on the patient (4). However, variability in its application has led to difficulty in drawing comparisons among studies and institutions (5). For example, when applied to a common elective orthopedic surgical procedure, like the tibial plateau leveling osteotomy (TPLO), use of the Cook system results in a broad range of complication rates (4.4–41.5%) (6–10) despite concurrent reports of consistently positive clinical outcomes (11).

While the Cook system provides general categories of complications (*minor, major, and catastrophic*) according to perceived clinical severity and outcome, the broad nature of these descriptors may contribute to subjective interpretation and variability in reported complication rates. The disparity between reporting high complication rates in procedures that have simultaneously high success rates has recently been termed “*complication rate inflation*” (12). This phenomenon may arise from the use of grading systems with fewer, broader categories require greater subjective interpretation of postoperative events. As a result, expected or clinically insignificant events may be misclassified as *complications*, leading to overestimation of complication rates.

To address these disadvantages, an adaptation of the Clavien-Dindo (aCD) system has recently been described and applied to veterinary orthopedic surgery (13). Unlike using three subjective categories as described by Cook et al. (4), the aCD system provides greater categorical resolution with five defined grades. It also explicitly distinguishes complications from two separate postoperative outcome measures: ‘*sequelae*,’ which are expected postoperative occurrences, and ‘*failures-to-cure*,’ in which surgery is executed successfully but clinical signs persist (13). By defining each grade according to the type of therapeutic intervention required to manage the complication, the aCD system aims to increase objectivity and interpretability, thereby improving correlation with clinical outcomes (1–3, 13). Research has demonstrated that when comparing the reliability of the two grading systems for events classified as true complications (excluding other non-complication outcomes like *sequelae* or *failures-to-cure*), the aCD system demonstrated a superior interobserver agreement (13).

The decision of which grading system to use can affect complication rates, and the extent to which the aCD system may alter complication reporting relative to the Cook system in a prospective clinical setting remains unknown. Given these gaps, there is a need to evaluate how the different systems may alter the reported complication rates of various surgical procedures. Therefore, the objective of this study was to prospectively record and compare the nature and frequency of postoperative complications in a population of dogs undergoing TPLO using both the Cook and aCD systems. We hypothesized that there would be no difference in the frequency or severity of reported complications between the two grading systems.

## Materials and methods

### Inclusion criteria

All client-owned dogs that underwent a TPLO for cranial cruciate ligament disease at the University of Missouri Veterinary Health Center between December 1, 2023, and January 1, 2025, were screened for enrollment. Inclusion criteria consisted of dogs undergoing a standard TPLO without adjunctive or modified procedures, and at least one postoperative data point from any source to document potential complications. Dogs were excluded if any prior surgery had been performed on the same stifle or if no postoperative information was available from any source after surgery. Bilateral cases undergoing staged TPLOs were included, and each stifle was analyzed as an independent surgery. Owner consent was obtained for the review of institutional and referring veterinary medical records and the completion of a standardized postoperative questionnaire (Supplementary material 1).

### Signalment and clinical information

Recorded patient data included age at surgery, breed, reproductive status, body weight, duration of lameness, affected limb, and stifle evaluation method (arthrotomy or arthroscopy). The extent of cranial cruciate ligament disease (complete or partial rupture) and the presence of meniscal injury were also documented. All TPLO procedures were performed by board-certified veterinary surgeons or surgical residents under the direct supervision of a board-certified surgeon. Surgeries were performed according to the institution’s standardized TPLO protocol, including standard blade sizes and soft tissue closure techniques. TPLO fixation was performed using commercially available implants from either the Arthrex (Arthrex Vet Systems, Naples, FL, USA) or Synthes (DePuy Synthes Vet, West Chester, PA, USA) TPLO systems. Perioperative analgesic protocols were determined by the attending anesthesiologist and primary case clinician, and perioperative antibiotic use was based on clinician preference.

### *A priori* power analysis

An *a priori* power analysis was performed using R (14) to determine the minimum sample size required for the paired comparison. Assuming a baseline complication proportion of 30% and a minimum detectable absolute difference of 20% between grading systems, fifteen surgeries were sufficient to achieve 80% power at α < 0.05. Because enrollment exceeded this threshold, the study was considered adequately powered.

### Postoperative event identification

For the purposes of this study, a *potential complication* was defined as any deviation from an expected postoperative course that prompted concern by the client, referring veterinarian, or surgeon. The incidence and classification of the potential complications were then compared between grading systems (Cook versus aCD).

A standardized protocol for documenting postoperative case progression and identifying potential complications was followed. Four postoperative data collection time intervals were established *a priori*: 10–28 days, 42–70 days, 4–8 months, and ≥8 months. The type of evidence collected at each time interval was dependent on if the patient was evaluated: cases seen postoperatively by the referring veterinarian or at the Veterinary Health Center included medical record review in addition to owners completing a questionnaire. Those patients not re-examined at the referring veterinarian’s office, or the Veterinary Health Center, were assessed only by owner questionnaire completed by phone or email. The questionnaire consisted of a standardized survey assessing incisional appearance, pain, and lameness using visual analogue scales (15), as well as any additional medications, treatments, hospital visits, or surgical interventions required. Additionally, referring veterinarians were contacted by phone at least twice (between 1–7 months and ≥ 8 months postoperatively), and any concerns or potential complications were recorded. Any patient without postoperative follow-up data was excluded.

All cases underwent orthogonal preoperative, immediate postoperative, and 8- and/or 12-week postoperative stifle radiographs. Radiographs were evaluated for implant integrity, bone and soft tissue pathology, and evidence of radiographic bony union defined by osseous bridging of the osteotomy and new bone formation along the caudal cortex. Incisional health was assessed using specific descriptors adapted from CDC-defined criteria for surgical site infections (16, 17). A wound was classified as *infected* if any of the following were present: purulent discharge, abscess or fistula, deep-incisional dehiscence, fever, ≥1 clinical sign of localized inflammation (pain, swelling, redness, heat), or positive bacterial growth from an aseptically collected sample.

### Postoperative event grading

Any potential complication at any given time point was considered an individual data point subject to grading. A single investigator (small animal surgery resident who participated in the clinical management of some study patients) graded all potential complications following a review of the methodologies described by Cook et al. (4) and Barrett et al. (13), as well as completing a formal pre-study training exercise to ensure familiarity with the aCD system (2, 13). The Cook and aCD grading systems are outlined in Tables 1, 2 respectively (4, 13). Each reported potential complication was graded independently using both systems. Classification of sequelae and failures-to-cure followed the published definitions described by Sink et al. (2) and Barrett et al. (13). Sequelae were defined as expected and unavoidable postoperative events inherent to the procedure that did not represent deviations from the anticipated postoperative course. Failures-to-cure were assigned only when the intended surgical objective was not achieved despite appropriate execution of the procedure. Appropriate execution was determined by review of the complete medical record, serial postoperative radiographs, and surgeon documentation to confirm that no technical or postoperative complication was identified that could reasonably account for the persistent clinical finding.

**Table 1:** Cook complication grading system.

System	Grade/category	Definition
Cook	Minor	Complication not requiring additional surgical or medical treatment to resolve (e.g., bruising, seroma, minor incisional complications).
Cook	Major	Complication requiring additional treatment based on standard of care, including: (1) surgical intervention, or (2) medical treatment.
Cook	Catastrophic	Complication or associated morbidity resulting in permanent unacceptable function, directly related to death, or leading to euthanasia.

Postoperative complications classified as minor, major, or catastrophic according to the level of intervention required and associated clinical outcome.

**Table 2:** Adapted Clavien–Dindo (aCD) complication grading system.

System	Grade/category	Definition
aCD	Grade I	No treatment required; no deviation from routine postoperative care. Permitted treatments include antiemetics, analgesics, anti-inflammatories, antibiotics, and physiotherapy.
aCD	Grade II	Deviation from normal postoperative course requiring outpatient treatment (e.g., pharmacologic therapy, close monitoring, or minor procedures not requiring general anesthesia).
aCD	Grade III	Complication requiring surgical, arthroscopic, or radiographic intervention; unplanned hospitalization; or management under anesthesia.
aCD	Grade IV	Life-threatening complication requiring ICU-level care, associated with potential permanent disability or organ dysfunction.
aCD	Grade Va	Death secondary to a perioperative complication.
aCD	Grade Vb	Euthanasia due to poor prognosis or inability to manage the complication.
aCD	Sequelae	Events inherent and unavoidable following the procedure.
aCD	Failures-to-cure	Failure to achieve the intended outcome of the surgery despite appropriate execution.

Postoperative complications classified according to grades I–V based on the level of therapeutic intervention required. Sequelae and failures-to-cure were recorded as outcome measures and not considered complications.

If a patient had multiple potential complications at different time points, only the most severe, as determined by both systems, was used for subsequent comparative analysis, allowing comparison of both complication frequency and severity between grading systems.

### Statistical analysis

Data were reported as mean ± standard deviation for independent variables and as median (range) for non-independent variables. Outcomes were dichotomized for both systems as either ‘complication present’ or ‘no complication,’ with potential complications classified as ‘sequelae’ or ‘failures-to-cure’ using the aCD system subsequently coded as ‘non-complications’. Paired complication rates were compared using a McNemar’s *χ*^2^ test with significance set at α < 0.05. All statistical analyses, including descriptive statistics and McNemar’s *χ*^2^ test, were performed using R (14).

## Results

### Patient demographics

Eighty-five TPLO surgeries in seventy-nine dogs met the inclusion criteria. Most dogs were female spayed (64.6%, 51/79), followed by male castrated (31.6%, 25/79), female intact (2.5%, 2/79), and male intact (1.3%, 1/79). The most common breeds were mixed-breed dogs (35.4%, 28/79), Labrador Retrievers (15.2%, 12/79), and American Staffordshire Terriers (12.7%, 10/79). Full breed distribution is shown in Table 3. The median age at surgery was 5.5 years (range, 1–12 years), median body weight was 31.0 kg (range, 7.9–66 kg), and median lameness duration was 4.75 months (range, 0.25–48 months).

**Table 3:** Breed distribution of the study population (*n* = 79).

Breed	Number of dogs, *n* (%)
Mixed breed dog	28 (35.4)
Labrador Retriever	12 (15.2)
American Staffordshire Terrier	10 (12.7)
Golden Retriever	6 (7.6)
German Shepherd	5 (6.3)
Australian Shepherd	3 (3.8)
Rottweiler	2 (2.5)
Border Collie	2 (2.5)
Beagle	2 (2.5)
Pembroke Welsh Corgi	1 (1.3)
Mastiff	1 (1.3)
Belgian Malinois	1 (1.3)
Whippet	1 (1.3)
Samoyed	1 (1.3)
Bichon Frise	1 (1.3)
Great Dane	1 (1.3)
English Bulldog	1 (1.3)
Boxer	1 (1.3)

Distribution of breeds included in the study population, reported as number of dogs and percentage of total cases.

### Surgical findings

Surgical laterality was evenly distributed (right, 51.3%; left, 48.2%). Cranial cruciate ligament pathology consisted of complete tears in 75.3% of stifles (64/85) and partial tears in 24.7% (21/85). Stifles were assessed by arthrotomy in 81.2% (69/85) and arthroscopy in 18.8% (16/85). A medial meniscal tear was present in 64.7% of stifles (55/85).

### Follow-up documentation

Owner survey responses were more concentrated within the first 60 postoperative days. Examinations performed at the Veterinary Health Center and primary veterinary clinics were more evenly distributed throughout the postoperative period, with some extending beyond 500 days. The median time to last follow-up for all surgeries was 286 days (range, 20–602 days; SD, 196.1 days).

A total of 100 potential postoperative complications were recorded. Most potential complications occurred within the first 3 postoperative weeks (66/100, 66%), with additional events identified between 42–70 days (13/100, 13%), 4–8 months (12/100, 12%), and ≥8 months (9/100, 9%). Most potential complications identified in the first 3 weeks were captured via owner questionnaire, whereas clinician examinations identified potential complications across all postoperative intervals.

**Figure 1:**
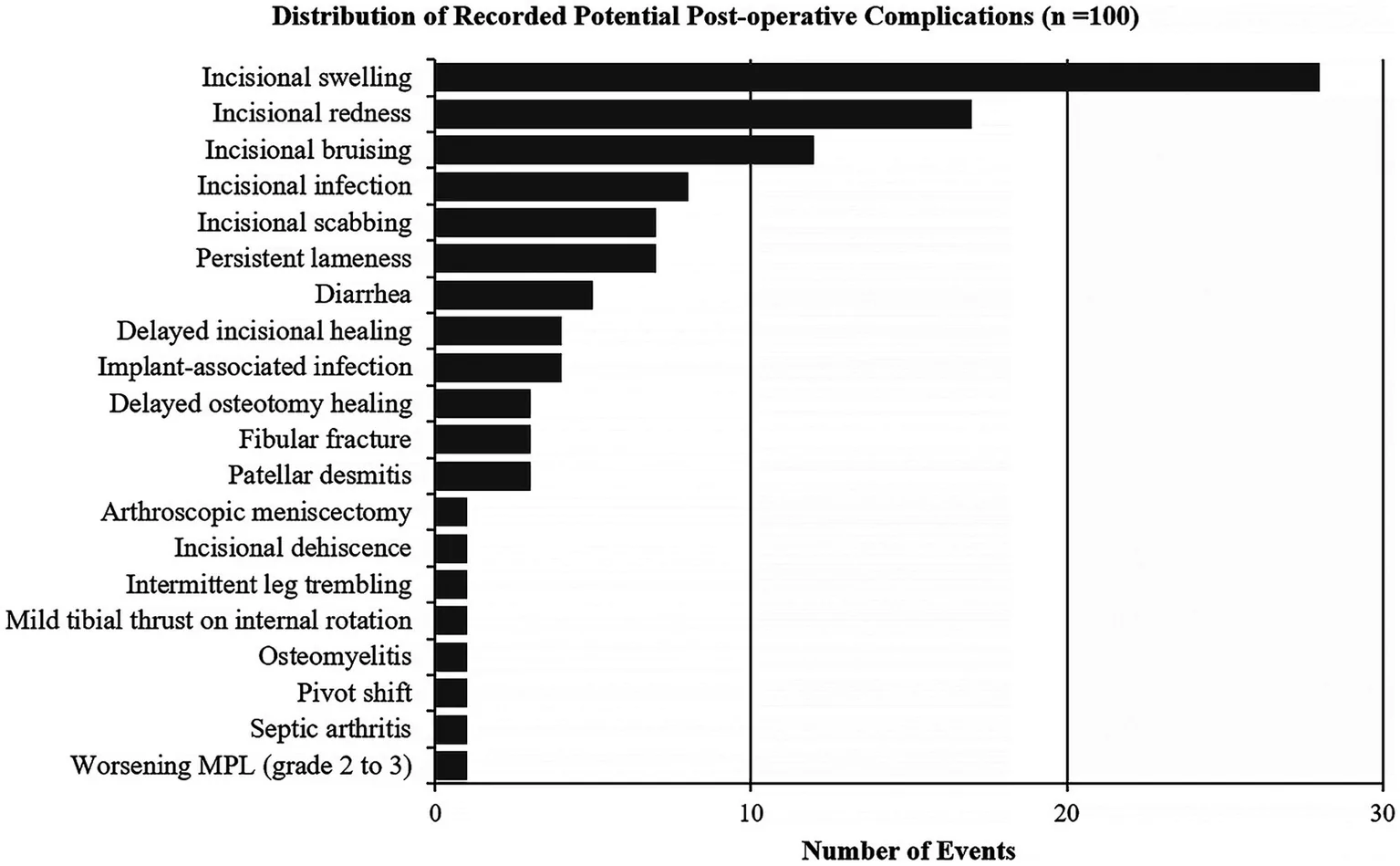
Distribution of potential post-operative complications (*n* = 100). Frequency of recorded post-operative complications in the study population. Incisional complications were most common, followed by less frequent events including delayed osteotomy healing, fibular fracture, and patellar desmitis.

Of all postoperative events identified as potential complications, concerns regarding the appearance of the surgical incision were most common (Figure 1). Fifty-five (55/100) specifically utilized the descriptors “swelling,” “redness,” and/or “bruising” when documenting concern about the incision. In decreasing frequency, other potential complications included incisional infection (9), persistent lameness (7), diarrhea (4), implant-associated infection (3), delayed incisional healing (3), delayed osteotomy healing (2), fibular fracture (2), and patellar desmitis (2). Single instances (*n* = 1) of secondary meniscal tearing, septic arthritis, development of a pivot shift, medial patellar luxation, development of tibial thrust, and osteomyelitis were also recorded.

### Complication classification

Using the adapted Clavien–Dindo (aCD) system, the overall complication rate was 28.2% (24/85), consisting of grade I (12.9%, 11/85), grade II (9.4%, 8/85), and grade III (5.9%, 5/85); no grade IV or V complications were identified (Table 4). Twenty-one cases (24.7%) were classified as sequelae, and one case (1.2%) was classified as a failure-to-cure. Table 5 demonstrates examples of how the same postoperative events were classified differently according to the two systems.

**Table 4:** Postoperative complication rates following TPLO using the Cook and adapted Clavien–Dindo (aCD) systems.

Classification system	Category	Percentage (%)
Cook system	**Overall**	**54.1**
	Minor	40.0
	Major	14.0
	Catastrophic	0.0
aCD system	**Overall**	**28.2**
	Grade I	12.9
	Grade II	9.4
	Grade III	5.9
	Grade IV	0.0
	Grade V	0.0
	Sequelae	24.7
	Failure-to-cure	1.2

Overall complication rates and distribution by grade following tibial plateau leveling osteotomy (TPLO), reported as percentages of total procedures (*n* = 85). Complications were categorized as minor, major, or catastrophic using the Cook system and grade I–V using the aCD system. Sequelae and failure-to-cure were recorded as non-complication outcomes under the aCD system. Bold text indicates overall complication rates for each classification system.

**Table 5:** Representative examples of postoperative event classification using the adapted Clavien–Dindo (aCD) and Cook systems following TPLO.

Postoperative event	aCD system	Cook system
Incisional redness, swelling, bruising	Sequelae	Minor
Post-operative diarrhea	Grade I	Minor
Delayed osteotomy healing requiring prolonged activity restriction	Grade II	Minor
Incisional infection	Grade II	Major
Implant associated infection	Grade III	Major
Persistent lameness—intermittent NSAID use	Failure-to-cure	Major

Representative examples of postoperative events illustrating differences in classification between the adapted Clavien–Dindo (aCD) and Cook grading systems following TPLO. Sequelae are expected postoperative outcomes and failures-to-cure represent persistent failure to achieve the intended surgical outcome despite appropriate execution of the procedure; neither is considered a complication under the aCD system. NSAID, nonsteroidal anti-inflammatory drug.

Using the Cook classification system, the overall complication rate was 54.1% (46/85), including minor (40.0%, 34/85), major (14.0%, 12/85), and catastrophic (0%) complications (Table 4). After dichotomizing each postoperative potential complication as a ‘true complication’ or ‘no complication’ according to the individual systems’ definitions, McNemar’s *χ*^2^ test demonstrated a significant difference in complication rates between systems (*p* < 0.001). Twenty-two events (26%) classified as a complication were categorized as *sequelae* or *failure-to-cure* under the aCD system. With respect to the nature of graded complications as assessed by the two systems, more advanced complications (Grades III-V using the aCD system, and ‘major’ and ‘catastrophic’ using the Cook system) were grouped and compared. The Cook grading scheme identified 14% of events as either ‘major’ or ‘catastrophic’ whereas the aCD system only identified 6% as grade III-V. A McNemar’s *χ*^2^ test demonstrated a significant difference between the two systems (*p* < 0.02).

## Discussion

The objective of this study was to compare the frequency and nature of potential postoperative complications in dogs undergoing a common elective orthopedic procedure using two different grading systems. The null hypothesis was that there would be no difference in complication rates reported by the two systems. However, our results demonstrate that the Cook system classified nearly twice as many potential complications as true complications when compared to the aCD system. Similarly, the Cook system identified significantly more advanced complications than the aCD system. Therefore, our null hypothesis was rejected.

This discrepancy was driven primarily by the differences in how each system categorizes postoperative events (Table 5). Specifically, the Cook system classifies sequelae and failures-to-cure as minor and major complications, respectively, whereas the aCD system distinguishes these as non-complication outcome categories. Importantly, classification of failures-to-cure remains procedure dependent and may occasionally be difficult to distinguish from true complications. Nevertheless, the adapted Clavien–Dindo system intentionally categorizes failures-to-cure as separate outcome measures because they reflect failure to achieve the intended surgical objective rather than a deviation from the expected postoperative course.

Consequently, the Cook system resulted in a substantially higher overall complication rate (54%) compared to the aCD system (28%). Similarly, when comparing the most severe complication categorizations identified in this population, the Cook system yielded a 14% major complication rate, compared to only a 6% rate of grade III complications using the aCD system.

It is well established that the selection of a classification system influences the reported incidence and nature of postoperative complications (1, 18). A lack of consensus regarding definitions and inconsistent application of widely accepted complication classification systems hinders meaningful comparison across studies (1, 5). Notably, the only systematic review evaluating the safety and efficacy of the TPLO procedure compared to alternative techniques was negatively impacted by subjective outcome measures, including postoperative complication grading (5). This variability may explain the wide range of reported complication rates, despite consistent evidence confirming that the TPLO is a safe and effective procedure (5, 11).

These findings are consistent with the data reported here. The overall postoperative complication rate was 28% using the aCD system, with a 6% risk of grade III complications, representing the highest level of severity observed. Interestingly, these results are similar to reported postoperative complication rates for a comparable human procedure, high tibial osteotomy, with an overall complication rate of 0–26% (18). Qualifying the relative risk of postoperative complications is subjective and inherently influenced not only by the frequency of complications, but also by their severity and clinical consequences. Therefore, a high frequency of less-severe complications (grade I or II in the aCD system, “minor” in the Cook system) may not be perceived as *high risk*, whereas a moderate frequency of more severe complications (grade III or higher in the aCD system; “major” in the Cook system), which require surgical or invasive intervention, may be considered *high risk* procedures. For example, surgical correction of adult spinal deformities in humans has an overall complication rate of 55%, with “major” complications occurring in 19% of cases (19). These procedures have been described as a group of techniques that pose “enormous” surgical risk (19, 20). Although the consequences of postoperative complications differ between neurosurgical and appendicular limb orthopedic procedures, the numeric values are similar to those reported for this population of TPLO patients when applying the Cook system, suggesting the TPLO is a risky procedure.

This study has several limitations. First, all potential postoperative complications were graded by a single investigator who participated in the care of some study patients. Although this familiarity may have influenced grading decisions, all events were classified according to the published Cook and aCD methodologies following formal pre-study training. This introduces the potential for observer bias; however, this approach is consistent with previously published TPLO complication studies using the Cook and aCD systems, some of which demonstrate excellent intraobserver reliability (7, 10, 11, 13). Second, although data were collected prospectively, the use of owner questionnaires introduces inherent reporting bias, as early, minor, or subjective abnormalities are more likely to be captured by owners than by clinicians. This reliance on non-medically trained individuals may affect the classification of postoperative events as potential complications. Third, neither the Cook nor the aCD classification systems fully eliminate subjectivity, particularly regarding the interpretation of event severity, clinical relevance, and categorization of borderline postoperative findings.

In conclusion, our findings demonstrate that complication rates are directly influenced by the grading system used. Systems, such as the adapted Clavien-Dindo system, which provide greater categorical resolution, may offer a more accurate representation of surgical risk. Importantly, distinguishing expected postoperative outcomes from true complications has a substantial impact on reported complication rates. Ultimately, the classification system chosen shapes the interpretation of surgical safety.

## Data Availability

The raw data supporting the conclusions of this article will be made available by the authors, without undue reservation.

## Supplementary material

SUPPLEMENTARY TABLE 1 Standardized owner survey questionnaire completed by telephone or email to collect postoperative outcome data following tibial plateau leveling osteotomy (TPLO).
