# Supplementary material for: Comparison of two postoperative complication grading systems following an elective orthopedic procedure in dogs
Source: Front Vet Sci. 2026 Aug 12;13:1867094. doi: 10.3389/fvets.2026.1867094 (PMC13506293; doi:10.3389/fvets.2026.1867094)
Supplement: Supplementary file 1 [file Table_1.DOCX]

| Animal Name:  Medical ID #: | Date of Survey Completion: | Date of Surgery: |
| --- | --- | --- |

Surgery: TPLO

Operated Side:	Left	Right

1. When was your pet’s surgery?

- 10-21 days ago
- 42-70 days ago
- 4-8 months ago
- 8 months +

2. Was there any of the following incisional concerns during the first 3 weeks after surgery:

- Bruising
- Swelling
- Redness
- Purulent discharge
- None

3. Rate your pet’s current pain level:

4. Rate your pet’s current lameness level:

5. Were there any additional trips to a veterinary hospital besides the planned appointments (for the incision check and recheck x-rays)?

- Yes
- No

6. If yes to #5, were there additional medications prescribed, treatments recommended, or surgical interventions?
7. Any other concerns or complications at home to report?
